# Clinical commentary of the evolution of the treatment for chronic painful mid-portion Achilles tendinopathy

**DOI:** 10.1590/bjpt-rbf.2014.0117

**Published:** 2015-10-06

**Authors:** Håkan Alfredson

**Affiliations:** 1 Department of Community Medicine and Rehabilitation, Sports Medicine Unit, Umeå University (UMU), Umeå, Sweden; 2 Pure Sports Medicine Clinic, London, UK; 3 The Institute of Sport Exercise & Health (ISEH), University College London Hospitals (UCLH), London, UK

**Keywords:** rehabilitation, tendinosis, eccentric training

## Abstract

The chronic painful Achilles tendon mid-portion was for many years, and still is in many countries, treated with intratendinous revision surgery. However, by coincidence, painful eccentric calf muscle training was tried, and it showed very good clinical results. This finding was unexpected and led to research into the pain mechanisms involved in this condition. Today we know that there are very few nerves inside, but multiple nerves outside, the ventral side of the chronic painful Achilles tendon mid-portion. These research findings have resulted in new treatment methods targeting the regions with nerves outside the tendon, methods that allow for a rapid rehabilitation and fast return to sports.

## Background

Chronic painful mid-portion Achilles tendinopathy is a relatively common condition among recreational and elite athletes, but it is also seen in non-active individuals. It is most common between the age of 36 and 60 and very rare among individuals younger than 25 years. The etiology is unknown, but an altered lipid profile with high cholesterol levels has been found in 1/3 of the patients^1^. Excessive dorsiflexion in the ankle joint^2^ and low calf muscle strength have also been suggested as possible etiological factors. Conservative treatment with different loading regimens is the first line of treatment, and if that fails, surgical treatment is instituted. For surgical treatment, intratendinous revision via tenotomy followed by 4-6 months of rehabilitation has been the most commonly used procedure worldwide.

The purpose of this clinical commentary is to show how the results of research on the basic science for this condition has resulted in a completely new treatment strategy with major advantages for the patients.In the 1990s, the Sports Medicine Unit in Umeå, Sweden, as in most other countries, used intratendinous revision surgery to treat patients with chronic painful mid-portion Achilles tendinopathy. Patients not responding to conservative management were treated with open surgery, including excision of macroscopically abnormal tendon tissue via a central longitudinal tenotomy, followed by immobilization in a cast for 2-6 weeks, with a total 4-6 months rehabilitation period.

By coincidence, our group at the Sports Medicine Unit in Umeå tried a modified version of the Stanish et al.^3^ model for eccentric calf muscle training. We used a level of loading that was causing pain in the tendon during the exercise and the exercises were done at a slow pace, in contrast to pain-free exercises and gradually increased speed. We got surprisingly good clinical results. To achieve good clinical results after applying painful heavy loading on a chronic painful Achilles tendon was completely opposite to previous thinking around treatments of chronic painful tendons, and the good clinical results^4^led to research into the pain mechanisms involved in chronic painful mid-portion Achilles tendinopathy.

## Painful eccentric calf muscle training

Our group designed an eccentric training regimen modified from the Stanish et al.^3^ model to be tried on patients suffering from chronic painful mid-portion Alfredson H

Achilles tendinosis. The training program included eccentric training over a step - 3x15 reps with straight and flexed knee performed 2 times/day, 7 days/week, for 3 months^4^. The method was tested in scientific studies^4^^-^ 6, and the overall results were very good, with around 80% satisfied and pain-free patients. After a while, we found out that high-level athletes, especially runners and jumpers who wear spiked shoes, did not have such good results with this treatment. Also, we found it to be of significant importance to establish that the patients had a correct diagnosis before the start of treatment. A partial rupture has to be excluded, because using eccentric training on a partially ruptured Achilles can further damage the tendon, possibly causing a lengthening of the tendon, that is known to be very difficult to treat.

Ultrasound follow-ups were performed on patients with chronic Achilles tendinopathy and very interestingly showed that in the successfully treated patients the Achilles tendon thickness had decreased over time, and the structure looked more normal sonographically^7^. Consequently, it appeared that painful eccentric calf muscle training had the potential to remodel the tendinosis tendon. From these research projects, where high painful loads were applied to the thick and painful Achilles tendons, we also learned that tolerating these high eccentric loads clearly show that the Achilles tendinosis tendon is not what had previously been thought: a so-called degenerative and weak tendon. Instead, it might very well be a strong tendon!

## New research on tendon histology and imaging

**Figure 1:**
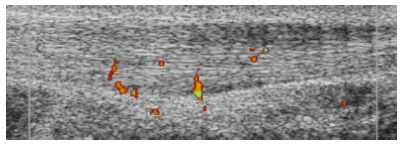
Ultrasound and Doppler examination showing a thickened Achilles tendon mid portion with irregular structure and high blood flow outside and inside the ventral side of the Achilles tendon.

We could not explain the background to the good clinical results achieved with painful eccentric training, and this led to extensive research together with Professor Sture Forsgren's group at the Anatomy Department and Dr Lars Öhberg at the Department of Radiology at Umeå University. Using ultrasound+Doppler, we found high blood flow inside and outside the ventral side of the Achilles tendon mid-portion in patients with chronic painful mid-portion Achilles tendinopathy, but not in normal Achilles tendons^8^ (Figure 1). In a following study, ultrasound+Doppler-guided biopsies were taken from the region with high blood flow inside and outside the Achilles mid-portion in patients with chronic painful tendinosis. Immune-histochemical analyses showed nerves in close relation to blood vessels outside the tendon, but very few nerves inside the tendon^9^. 

An interesting observation was that these were mainly sympathetic nerves, but also a few sensory nerves^9^. To try to trace the pain, ultrasound+Doppler-guided injections of small volumes of the local anesthetic xylocain+Adrenaline were administered, targeting the regions with high blood flow outside the tendon. This temporarily cured the tendon pain^10^. These findings clearly indicated that the pain in mid-portion Achilles tendinopathy comes from the nerves located on the ventral side of the Achilles, and that the nerves can indirectly be found by using ultrasound+Doppler to find the regions with high blood flow (blood vessels with accompanying nerves).

## Ultrasound+Doppler-guided sclerosing polidocanol injections

The new research findings related to the reduction of pain at the regions of highest blood flow led to the invention of a new treatment method: ultrasound+Doppler-guided injections of the sclerosing substance polidocanol, targeting the regions with high blood flow and nerves outside the tendon. This type of treatment showed good clinical results with significantly lowered pain scores (VAS) during Achilles tendon loading activity in pilot studies and in a randomized placebo-controlled study^11^^,^ 12. Ultrasound+Doppler 2-year follow-ups of patients treated with sclerosing polidocanol injections showed decreased tendon thickness and improved structure (less irregular structure with less hypo-echoic regions) over time^13^, indicating a high potential in the soft tissues outside the ventral side of the Achilles tendon. The limitations with ultrasound+Doppler-guided polidocanol injections are that it is technically demanding, having a relatively long learning curve, and that often multiple^4^^,^ 5 injection treatments are needed.

## Ultrasound+Doppler-guided mini-surgical scraping

To try to overcome the problems with the technically demanding polidocanol injection treatment, our group at the Sports Medicine Unit in Umeå invented a mini-surgical scraping treatment. Guided by the ultrasound+Doppler findings, a minor surgical procedure is performed under local anesthesia. Using a longitudinal lateral mini (1 cm) incision, the ventral side of the tendon is scraped in the regions with high blood flow and nerves^14^^,^ 15. This is a one stage and more radical approach to interfere with the nerves accompanying the blood vessels on the ventral side of the Achilles. Because there is no intratendinous treatment associated with this procedure, a relatively fast (4-6 weeks) rehabilitation can be used. The patients start walking with full weight bearing the first day after the operation and rapidly progress to functional tendon loading. There is no specific eccentric training regimen, but instead, there is a general build-up of training, depending on the requirements for the individual's tendon loading activity (high-level activity to non-activity). The clinical results are very good with significantly lowered pain scores (VAS) during Achilles tendon loading activity and return to pre-injury activity levels, without any major side effects. In the 1-2 year follow-up of these individuals, the results remain positive, and the use of this method has been increased. We now have operated on large numbers of patients with chronic AT and at different activity levels, including professional athletes^15^. For reasons still unknown, high-level athletes seem to do best after this procedure. Patients with low physical activity level showed good clinical results in about 70% of cases, while among high-level athletes the success rate was more than 90%^15^.

**Figure 2:**
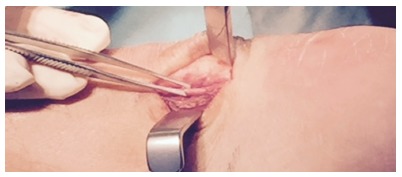
A thickened plantaris tendon located close to the thickened Achilles tendon mid-portion in a patient with chronic painful mid-portion Achilles tendinopathy+plantaris tendon involvement.

Recently, focus has been placed on the plantaris tendon, located in close relation to the medial Achilles. There seems to be a subgroup of patients suffering from chronic painful mid-portion Achilles tendinopathy, where a thickened plantaris tendon is involved^16^. These patients have both mid-portion Achilles tendinopathy with high blood flow on the ventral side of the tendon and a closely located plantaris tendon (demonstrated with ultrasound) with also a localized high blood flow (Doppler) on the medial side of the Achilles. These patients most often complain of having pain located on the medial side of the Achilles, where the medial soleus inserts. It is our observation that if the plantaris tendon is involved there is often a poor response to eccentric training. This can theoretically 

be explained by the fact that the plantaris tendon, known to be stronger and stiffer than the Achilles^17^, can cause a compression on the medial Achilles during the movements in the eccentric treatment regimen. When we noticed that the plantaris tendon could be involved, we changed the surgical technique from using a lateral incision to always using a medial incision to allow for an accurate evaluation of the relationship between the plantaris and Achilles tendons^18^ (Figure 2). If a plantaris tendon involvement is found, then the plantaris tendon is released proximally and distally, and 4-6 cm of its length are taken out. 

Very recently, we have noticed that there is a minor group of patients who have plantaris-related pain without also having mid-portion Achilles tendinopathy (verified with ultrasound+Doppler examination) (non-published data). These patients do very well after plantaris tendon removal alone. To study the innervation patterns of the plantaris tendon, immune-histochemical examinations were performed in a large number of plantaris tendons and surrounding fibrous connective and fat that were taken out from patients with mid-portion Achilles tendinopathy and plantaris involvement^18^. Although the results related to innervation patterns have not been published yet, they show that most sensory nerves are found in the peritendinous connective tissue between the Achilles and plantaris tendon, but in about 1/3 of the plantaris tendons, there are also nerves inside the plantaris tendon that may be a co-factor in the medial pain.

## Conclusions

Non-operative treatment with painful eccentric training is the first line of treatment for chronic painful mid-portion Achilles tendinopathy. Our research on the innervation patterns in patients with chronic painful mid-portion Achilles tendinopathy has shown that there are no (or very few) nerves inside the chronic painful Achilles tendon mid-portion. Instead, the nerves are found outside the ventral side of the tendon. This knowledge has led to the invention of a new mini-invasive surgical treatment, combined with a fast rehabilitation, to be used on the patients who have a poor result with eccentric training. With the use of this method, there is a very good chance of cure from chronic painful mid-portion Achilles tendinopathy and return to full activity, including Achilles tendon-demanding professional sports, within 4-6 weeks after surgery.
